# Defining Poverty as an Eligibility Requirement for Supportive Services

**DOI:** 10.1093/geroni/igab046.1656

**Published:** 2021-12-17

**Authors:** Allen Glicksman, Lauren Ring

**Affiliations:** 1 None, Philadelphia, Pennsylvania, United States; 2 Philadelphia Corporation for Aging, Philadelphia, Pennsylvania, United States

## Abstract

Deciding which individuals qualify as “poor” often depends on how each country or municipality defines the term ‘poverty’. In the United States, program eligibility is often tied to the Federal Poverty Level (FPL), using 100% of the FPL as a cut-off for receipt of services. However, research has shown that incomes of 200% of the FPL and higher are often needed to establish even minimum levels of economic security. Using data from an omnibus health study conducted in 2018 that included 1,581 persons ages 60+ who were asked about their health and service needs, we compared persons making 100% of the FPL or less to persons making 101%-199% and 200%+, respectively. Results show that poor health status and need for services among persons in the 101%-199% are similar to those with incomes less than 100% FPL, and significantly higher than persons with incomes at 200%+ of the FPL.

